# Flux measurements of the tricarboxylic acid cycle in the tumors of mice

**DOI:** 10.1093/lifemeta/load020

**Published:** 2023-05-24

**Authors:** Shiyu Liu, Jason W Locasale

**Affiliations:** Department of Pharmacology and Cancer Biology, Duke University School of Medicine, Durham, NC 27710, United States; Department of Pharmacology and Cancer Biology, Duke University School of Medicine, Durham, NC 27710, United States

**Measurements of metabolic reactions under physiological conditions have been a challenging problem. In a recent issue of *Nature*, Bartman *et al*. designed an isotope-labeling-based method to measure tricarboxylic acid (TCA) fluxes in normal tissue and tumors in mice. The method revealed that primary tumors exhibit lower TCA fluxes compared with normal tissue, consistent with current knowledge. They also found that solid tumors generally exhibit lower energy production rates.**

Traditional methodologies to study metabolism in animals provide a limited number of flux measurements, such as oxygen consumption, carbon dioxide release, and energy production [1]. Biochemical or pharmacological methods, on the other hand, typically require perturbations such as using specific reagents that can substantially disrupt normal metabolic activities. Additionally, indirect methodologies, like proteomics, do not reflect an actual metabolic state. Isotope-labeling methods deduce flux from labeling patterns measured by mass spectrometry or nuclear magnetic resonance. They have become the mainstream technology of flux analysis.

There are two main strategies of isotope-labeling methods: stationary and non-stationary. The stationary labeling strategy involves using the labeling ratio at the stationary level after long-time labeling to fit a linear model and deduce flux [2]. This approach requires fluxes to be constant during the labeling process. On the contrary, the non-stationary labeling strategy uses time-serial variant labeling values to fit an ordinary differential equation (ODE) model [3]. Although the non-stationary approach requires more preset parameters, a more complicated mathematical model, and more experimental data to achieve sufficient precision and accuracy, it can however reduce the labeling time needed and is appropriate when fluxes are time dependent [4].

The Warburg effect refers to the phenomenon where cells prefer anaerobic glycolysis for energy production rather than aerobic oxidation in the tricarboxylic acid (TCA) cycle even in the presence of oxygen [5]. Cancer cells are believed to have high energy demands but still choose the less efficient anaerobic glycolysis. Numerous hypotheses have been proposed to explain this phenomenon, including faster ATP production rate, limitations in proteome allocation, balancing the oxidative/reductive ratio, and the tumor microenvironment [5]. However, most studies are based on cultured cells, and those few *in vivo* studies sometimes lack absolute quantification of glycolytic and TCA flux.

**Figure 1:**
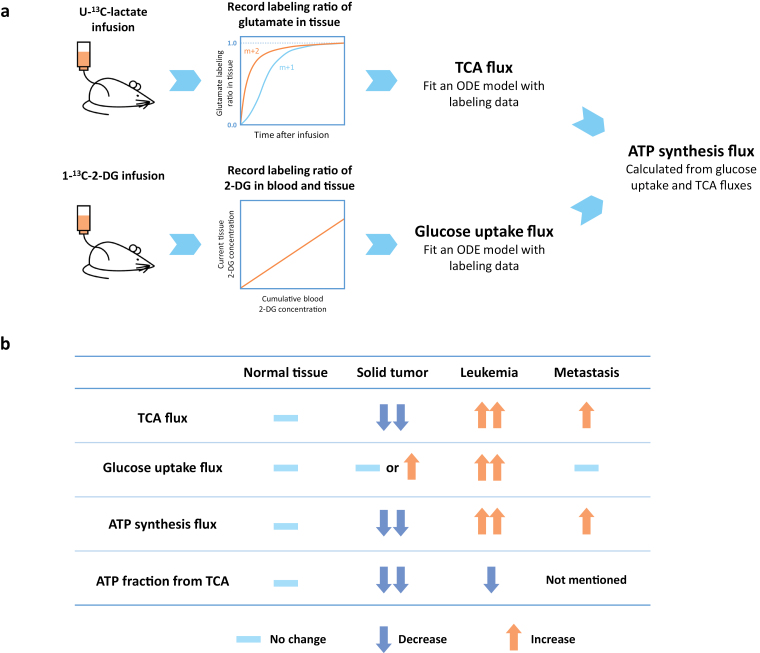
Measurement of TCA cycle and glucose uptake flux in normal tissue and different types of cancer. (a) Measurement protocol of TCA cycle and glucose uptake flux by non-stationary isotope-labeling and flux analysis. TCA and glucose uptake fluxes are utilized to calculate ATP synthesis rate. (b) Comparison of TCA cycle flux, glucose uptake, ATP synthesis, and ATP fraction from the TCA cycle across normal tissues and different types of ­cancer. 2-DG: 2-deoxyglucose, ODE: ordinary differential equations.

In their recent study, Bartman *et al*. optimized a flux analysis methodology that relies on the non-stationary isotope-labeling approach [6] (Fig. 1). They infused mice with different isotope-labeled substrates and collected samples at different time points, an extraordinary technical feat given the number of animals involved and the experimental precision needed, especially with regard to the time resolution, to achieve quantitative information. They then used an ODE model to fit the time-serial curve of labeling ratio to calculate target fluxes. Specifically, they used uniformly ^13^C labeled lactate (U-^13^C-lactate) to measure TCA fluxes and 1-^13^C labeled 2-deoxyglucose, an alternative to glucose that can be absorbed but not catabolized, to measure glucose uptake flux. They observed that solid tumors have similar or higher glucose uptake fluxes, but their ATP production rate is lower due to slower TCA fluxes. However, leukemia was found to have higher TCA fluxes than normal tissue and metastases have higher TCA fluxes than primary tumors. They also showed some evidence that tumors decrease ATP-consuming activities, such as synthesis of functional enzymes of normal tissue. These findings support the existence of a classical Warburg effect in physiological conditions, consistent with prior findings [7–9].

Their quantitative conclusions provide a perspective on the Warburg effect. One view is that cancer cells will have a higher energy demand than normal tissue due to their rapid proliferation. This study proposes another perspective: cancer cells can downregulate unnecessary biological activities to conserve energy for proliferation. This reduction of energy demand, combined with limited oxygen supply in tumor microenvironments, leads to the inhibition of TCA fluxes and the manifestation of the Warburg effect. However, in the case of leukemia where oxygen supply is not limited, cancer cells can still upregulate their metabolism to increase glucose uptake and produce ATP with a similar ratio of TCA fluxes as normal tissue.

There are several factors that require careful consideration when interpreting the results of this study. First, all fluxes are calculated by fitting ODE models with a few time-serial data points of labeling ratios, and the uncertainty and robustness of this fitting process, especially in mice, need to be evaluated more systematically in future studies. Second, the use of isotope-labeled lactate to measure TCA fluxes may lead to uneven uncertainty in tissues with different lactate preferences, and that decreases the precision and accuracy of flux results. Finally, the underlying cause of depressed TCA fluxes in cancer cells still requires further investigation and confirmation. For example, hypoxia is a major factor that will affect computed TCA rates as is the presence of necrotic cells, the non-tumor cells which in many cases comprise more than half of the tumor tissue. All aspects of tumor heterogeneity will influence these results, so collapsing the data into a single model for cancer cell metabolism is difficult. These are general limitations with bulk measurements that require spatial and single cell analysis in the future. Nevertheless, advanced flux analysis methodology based on isotope-labeling has significantly enhanced the ability of metabolism researchers to measure biological systems. These techniques have made it easier to obtain precise and robust *in vivo* measurements, and are providing unprecedented viewpoints and new insights into the field of metabolism studies.
